# Development of a thiol-ene microfluidic chip for CE-MS of peptides and proteins

**DOI:** 10.1007/s00216-026-06778-7

**Published:** 2026-09-10

**Authors:** Omid Rouhi, Jordan T. Aerts, Kamille C. Staack, Delaram R. Mikalsen, Stephan S. Keller, Mohammad Ramezannezhad, Nickolaj J. Petersen, Jörg P. Kutter, Kasper D. Rand

**Affiliations:** 1 Protein Analysis Group, Department of Pharmacy, University of Copenhagen, 2100 Copenhagen Ø, Denmark; 2 Pharmaceutical Analytical Chemistry Group, Department of Pharmacy, University of Copenhagen, 2100 Copenhagen Ø, Denmark; 3 National Centre for Nano Fabrication and Characterization, DTU Nanolab, Technical University of Denmark, 2800 Kgs., Lyngby, Denmark

**Keywords:** Microfluidics, Thiol-ene polymer, Capillary electrophoresis, Peptide and protein analysis, Mass spectrometry

## Abstract

Microfluidic capillary electrophoresis coupled with mass spectrometry (MCE-MS) has emerged as a promising platform for miniaturizing biomolecular analysis due to its low sample consumption, rapid separations, and potential for integration of multiple analytical functions on a single device. However, peptide and protein analysis with MCE-MS devices can be limited by electrospray ionization stability and surface adsorption. Here, we have developed a thiol-ene-based MCE device with an integrated electrospray emitter, fabricated using a double-replication molding approach. We improved the performance of the MCE-MS microchip by optimizing electrospray ionization stability and systematically evaluating surface modification strategies to minimize non-specific adsorption and improve separation repeatability. Different MCE channel surface coatings, including linear polyacrylamide and poly(ethylene glycol) methyl ether methacrylate (PEGMA), were investigated for their influence on peptide separations. Both coatings improved separation performance compared to unmodified devices, providing enhanced migration repeatability and reduced non-specific adsorption. PEGMA coatings provided the most balanced overall performance, combining comprehensive analyte compatibility with repeatable migration time and improved peak symmetry with narrower peak width. The optimized coated microchip was utilized for peptide mapping MCE-MS/MS analysis of pepsin-digested hemoglobin. PEGMA-coated chips enabled peptide identification within a separation window of approximately 60 s, achieving sequence coverages of 92.2% and 78.8% for the α- and β-chains, respectively. In addition, intact protein separations of insulin, β-lactoglobulin, and tropomyosin were demonstrated under acidic MCE-MS conditions, with repeatable migration times. These results highlight the potential of thiol-ene-based MCE-MS as a versatile platform for peptide and protein analysis.

**Graphical Abstract:**

**Figure:**
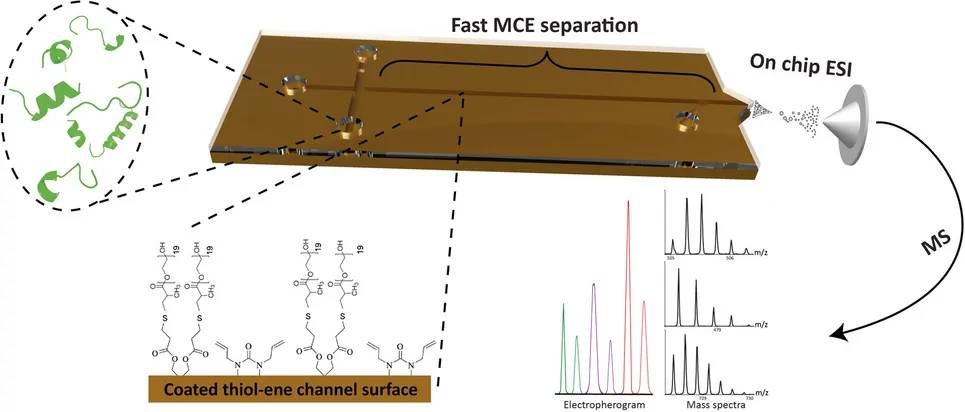

**Supplementary Information:**

The online version contains supplementary material available at https://doi.org/10.1007/s00216-026-06778-7.

## Introduction

Proteins and peptides are among the most important biomolecules, serving as critical analytical targets in biopharmaceutical development, disease research, and proteomics [1, 2]. Most drugs currently on the market target proteins, and a growing number of emerging therapies are protein-based [3–5]. Development of such complex biopharmaceuticals can be challenging, and they require extensive monitoring of unwanted modifications and degradation to ensure product quality [6]. Given these needs, sensitive and selective analytical techniques for protein analysis are crucial [7].

Reversed-phase liquid chromatography (LC) is widely used in protein analysis due to its strong separation power, automation capabilities, and straightforward coupling to various detection techniques, particularly mass spectrometry (MS) [8, 9]. LC-MS enables analysis of complex protein mixtures and facilitates the determination of protein mass [10], amino acid sequence, and post-translational modifications [11]. In addition, reversed-phase LC-MS is widely applied for quantitative analysis, providing accurate measurement of protein and peptide abundance across diverse biological samples [12–14]. It is also valuable for probing higher-order structure when combined with methods such as hydrogen/deuterium exchange mass spectrometry (HDX-MS) and crosslinking MS (XL-MS) [15–17]. While LC remains a very popular separation method, it faces significant challenges, particularly for large peptides and intact proteins. These include inefficient elution from the stationary phase, loss of higher-order structure due to the prolonged exposure to organic solvents, and poor selectivity [18]. Performance also declines at reduced temperatures, limiting its suitability for analytical approaches that require cold operating conditions, like HDX-MS [19–21]. These drawbacks have driven interest in alternative separation techniques like capillary electrophoresis (CE) [22].

CE separates analytes in fused-silica capillaries as a function of their charge and size, avoiding stationary phase interactions and facilitating efficient separation of large biomolecules [22–24]. In CE, analyte mobility not only is influenced by analyte charge and size but may also be affected by electroosmotic flow (EOF) of the background electrolyte (BGE). EOF originates from the charge at the capillary surface. In bare fused-silica capillaries, silanol groups can deprotonate, leaving negatively charged sites at the wall. Positively charged buffer species accumulate near this ionized surface, forming an electrical double layer. Upon application of an electric field, mobile ions in this double layer move toward the electrode of opposite polarity and carry the surrounding liquid with them, generating bulk flow. The magnitude and direction of EOF depend on the capillary wall surface charge density and the electrical potential at the surface in a given BGE at a given pH [25]. CE requires only a small sample amount [26] in low sample volumes and can maintain high separation performance at lower temperatures, potentially making it suitable for methods like HDX-MS [27, 28]. However, conventional CE platforms remain limited in their ability to integrate sample treatment/handling and additional analytical functionalities within a single workflow. These limitations have increased motivation to develop miniaturized CE systems capable of integrating multiple analytical steps.

Microfluidic capillary electrophoresis (MCE) offers a potential solution to these limitations by integrating with other microfluidic components for sample pretreatment or orthogonal separation, and provides additional benefits, including shorter analysis times and reduced reagent consumption [29, 30]. When coupled to MS detection, MCE-MS can achieve very efficient separation at high sensitivity, ideal for peptide and protein analysis [31]. A persistent challenge, however, is establishing a stable electrospray ionization (ESI) interface [32]. Various approaches have been explored to generate a stable nano-ESI for interfacing MCE with MS, including using a sheath flow, an external emitter, or a monolithically integrated ESI tip [33]. The ESI can also be generated directly from the microchip by forming the ESI at the chip corner. This design reduces complexity and eliminates the need for external pressure sources or additional capillary tips [34–36].

Recent MCE-MS developments have focused on enhancing separation efficiency by minimizing band broadening effects, non-specific adsorption, and incorporating sample preconcentration steps to boost sensitivity [29, 30, 37]. For instance, Carillo et al. performed native mass spectrometry using a commercial MCE-MS (ZipChip), to analyze the charge variant profiles of monoclonal antibodies, identifying up to 52 proteoforms in a single antibody drug product, including low-abundance species [38]. These advancements continue to broaden the applications of MCE-MS in various fields, including proteomics, peptidomics, and biopharmaceutical characterization [33, 39, 40].

While microfluidic devices show great promise, broader adoption is limited by the fabrication materials commonly used. Glass, despite its chemical resistance and optical clarity, is costly to process, especially for microdevices or low-volume production. Polymeric alternatives, such as poly(dimethylsiloxane) (PDMS), are inexpensive and transparent, and their simple replica molding fabrication enables rapid and low-cost prototyping. However, PDMS suffers from poor solvent resistance, leading to swelling in various solvents [41, 42]. Addressing these limitations, thiol-ene (TE) click chemistry has emerged as a robust and customizable polymerization process for microfluidic device fabrication [43–47]. The step-growth mechanism of TE polymerization enables rapid curing, minimizes deformation, provides low shrinkage stress, offers good solvent resistance, and exhibits minimal oxygen inhibition [48]. TE materials can be tuned by adjusting monomer composition, as in off-stoichiometry TE (OSTE) systems, to produce both rigid and flexible devices [49]. Moreover, they can polymerize without a photoinitiator, avoiding additives that can be toxic, costly, or cause yellowing [48, 50]. Additionally, heat treatment for up to 60 h at 200 °C further improves solvent compatibility by limiting solvent penetration [42].

Tähkä et al. fabricated TE-based MCE with laser-induced fluorescence detection and compared surface properties between stoichiometric TE (STE) and OSTE compositions [51]. They observed that the surface charge significantly depends on the composition ratio and the UV exposure dose. Under complete curing conditions, an EOF in a STE ratio was achievable, indicating a negative surface charge. Furthermore, the EOF almost doubled by ramping up the pH from 4.5 to 10, similar to a fused-silica capillary. In another study, a neutral polyacrylate copolymer coating was developed to modify the surface of the TE microchannel to enhance surface hydrophilicity and EOF stability and decrease protein adsorption. The coated microchannels were then successfully used for the separation of acidic and basic proteins [52]. To our knowledge, only a few studies have reported on TE-based MCE-ESI-MS until now [53, 54]. The first integrated on-chip emitter TE device demonstrated stable operation in both aqueous and non-aqueous conditions, enabling analysis of diverse analytes [53]. Notably, non-aqueous BGEs significantly enhance peptide separation. However, the authors observed that native uncoated TE surfaces suffer from non-specific adsorption, resulting in peak tailing [53].

Despite these advances, the potential of TE microchips for peptide and protein separations by CE–MS remains largely unexplored. Previous studies have mainly focused on small-molecule applications or basic demonstrations of concept. At the same time, challenges such as ESI stability, chip-to-chip repeatability, and non-specific adsorption at the channel surface remain unresolved. In particular, systematic evaluation of surface modification strategies to improve peptide compatibility has not yet been reported. Here, we developed a TE-based MCE-ESI-MS device and systematically investigated the impact of heat treatment, BGE composition, and various surface coatings on MCE-MS performance. We optimized conditions for efficient on-chip separation, ionization, and mass analysis of model peptides, complex peptide mixtures, as well as intact proteins.

## Materials and methods

### Chemicals and reagents

All chemicals and reagents were of analytical grade and used as received unless otherwise stated. Pentaerythritol tetrakis(3-mercaptopropionate) (PETMP), triallyl-1,3,5-triazine-2,4,6-trione (TATATO), hemoglobin (HB) from human, insulin, tropomyosin, β-lactoglobulin, angiotensin II (ATII), bradykinin (BK), leucine enkephalin (LE), methionine enkephalin (ME), substance P (SP), Glu-fibrinopeptide B (GFP), acrylamide, cysteamine, poly(ethylene glycol) methyl ether methacrylate (PEGMA), formic acid (FA), arginine, carnitine, HEPES, potassium phosphate monobasic (KH₂PO₄), octadecylamine (ODA), and basic aluminum oxide were purchased from Sigma-Aldrich/Merck Life Science A/S (Søborg, Denmark). Methoxy polyethylene glycol N-hydroxysuccinimide ester (mPEG-NHS, 20 kDa) was purchased from Nanocs (New York, NY, USA). Rhodamine B was purchased from Acros Organics (Geel, Belgium). Ethyl(2,4,6-trimethylbenzoyl)phenylphosphinate (TPO-L) photoinitiator was obtained from IGM Resins (Waalwijk, Netherlands). Methanol (MeOH), isopropanol (IPA), acetonitrile (ACN), and acetone were obtained from VWR Chemicals (Søborg, Denmark). Deionized water was purified using a milli-Q purification system. Sylgard 184 poly(dimethylsiloxane) (PDMS) elastomer kit was purchased from Dow Corning (Midland, MI, USA). SU-8 XFT 75 negative photoresist and SU-8 developer were obtained from Kayaku Advanced Materials (MA, USA). Immobilized pepsin agarose resin was obtained from Thermo Fisher Scientific.

### Microfluidic chip fabrication

Microfluidic chips were fabricated using a double-molding method as previously described by Lu et al. [54]. SU-8 master molds were used to cast PDMS molds, and then a STE mixture of PETMP and TATATO was poured into the PDMS molds and cured with UV light. SU-8 master molds were fabricated on 100 mm silicon wafers using SU-8 XFT 75 negative photoresist. Channel layouts were designed in CleWin. The mold consisted of two sequentially patterned SU-8 layers with target thicknesses of 200 µm and 50 µm. The first layer was spin-coated at 500 rpm for 30 s (200 rpm/s acceleration), followed by 700 rpm for 60 s (500 rpm/s acceleration), soft baked at 65 °C for 5 min and 110 °C for 45 min, exposed at 365 nm (400 mJ/cm^2^) using a µMLA Maskless Aligner (Heidelberg Instruments), and post-exposure baked at 65 °C for 5 min and 110 °C for 12 min. The second 50-µm-thick layer was deposited on top of the first layer using spin conditions of 1000 rpm for 30 s (500 rpm/s acceleration) and 3000 rpm for 60 s (1000 rpm/s acceleration), followed by soft bake (65 °C for 3 min, 110 °C for 7 min), exposure at 365 nm (260 mJ/cm^2^), and post-exposure bake (65 °C for 2 min, 110 °C for 8 min). The wafer was immersed in SU-8 developer for 45 min to remove uncured photoresist. After development, the completed master mold was hard baked at 120 °C for 10 h. All fabrication steps were carried out in the cleanroom facilities at the PolyFabLab, DTU Nanolab, Denmark.

PDMS molds were cast from the SU-8 master using a 1:9 ratio and cured at 80 °C for 2 h. Mixtures of thiol and ene monomers were cast into the PDMS molds and cured from both sides with a UV flood lamp (365 nm, 100 mW/cm^2^, Dymax 5000-EC, Dymax Corp., CT) for 5 s. The complementary slabs were aligned using a microscope and exposed to the same UV lamp for 5 min to complete the bonding process. To achieve good sharpness, the tip was trimmed manually using a scalpel. A chip holder featuring injection ports was drawn with computer-aided design software (Autodesk Inventor Professional 2025) and milled from PEEK by high-precision milling (Minitech 3, Minitech Machinery Corp., Norcross, GA, USA).

### Surface coating

Three surface modification strategies were evaluated to minimize non-specific adsorption and regulate EOF: cysteamine followed by mPEG-NHS, linear polyacrylamide (LPA), and PEGMA. For the cysteamine/mPEG-NHS coating, a 50 mg/mL cysteamine solution in milli-Q water with 0.2% v/v TPO-L was flushed into the channels, followed by two 33-s UV exposure separated by a 1-min cooling step between exposures. After rinsing with 50% MeOH (2 µL/min, 10 min) and drying with nitrogen, the channels were incubated with 100 mM KH₂PO₄ buffer (pH 7.5) containing mPEG-NHS (100 mg/1.2 mL) for 4 h in sealed petri dishes, then rinsed and dried again. LPA coating was performed by flushing a 10 mg/mL solution of acrylamide in milli-Q water with TPO-L, followed by five UV cycles of 2 × 33-s exposure and 1-min rest between exposures; after each cycle, the channels were rinsed with 50% MeOH (2 µL/min, 10 min) and dried. PEGMA modification was carried out by mixing PEGMA: milli-Q water: IPA (6:69:25% v/v), removing inhibitors by incubation with basic aluminum oxide, filtering, and adding TPO-L (0.2% v/v) before use. PEGMA coating was performed by flushing the previously described PEGMA solution into the chip channels followed by five UV cycles of 2 × 33-s exposure with 1-min rest between exposures. After each cycle, the channels were rinsed with 50% MeOH (2 µL/min, 10 min) and dried. The same UV flood lamp that was used in the fabrication process was utilized for all UV exposures of the coatings.

### Emitter tip coating

The ESI emitter was coated using a procedure modified from what we have reported previously [55]. A 2% PDMS solution containing 10% curing agent was prepared in IPA by ultrasonication for 15 min (solution A). Separately, a 4% octadecylamine (ODA) solution in IPA was prepared under ultrasonication at 40 °C (solution B). Immediately prior to coating, solutions A and B were combined to obtain the final coating mixture. To apply the coating to the chip’s emitter, a flow of 0.5 µl/min (50% MeOH) was directed to the side channel to prevent the coating solution from entering the channel. Then the tip was submerged in the coating solution for 3 min and immediately placed in an oven at 45° C for 2 h. After curing, the tip was thoroughly cleaned by fully immersing it in milli-Q water, then in acetone, and finally in milli-Q water to remove any unreacted PDMS and ODA.

### Operating the MCE-MS chip

The microchips were interfaced with two different types of mass spectrometers: an LTQ Orbitrap Velos (Thermo Fisher) using a Nanospray Flex ion source, and a Synapt G2-Si Q-TOF (Waters) using a lab-built interface. For the LTQ Orbitrap Velos, the chips were prepared by attaching glass cylinders (2 × 6 mm) to the inlet holes to serve as reservoirs. Later, a chip holder with integrated reservoirs was designed. LTQ Orbitrap Velos operated in positive mode with a m/z range of 150–2000, a resolution of 30,000, and microscan 1. The Waters Synapt G2-Si Q-TOF mass spectrometer operated in positive ion mode and resolution mode, with an m/z range of 50–2000, a scan rate of 1/s, a source temperature of 90 °C, and a sample cone voltage of 30 V.

**Fig. 1:**
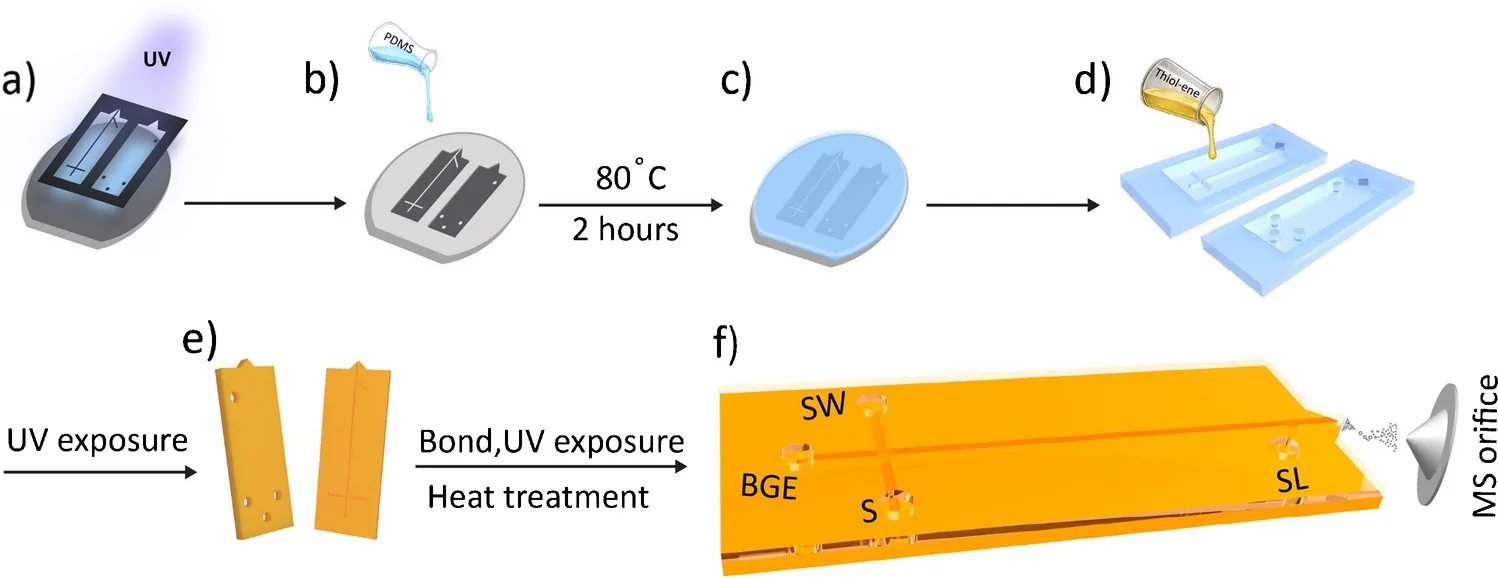
Fabrication and design of the thiol-ene (TE) based MCE-MS microchip. **a** Photolithography on a silicon wafer utilizing SU-8 (first mold). **b** PDMS casting. **c** Cured PDMS layer on top of the silicon wafer (second mold). **d** TE casting and polymerization. **e** TE slabs featuring microchannels and inlets. **f** MCE schematic with microchip design and reservoirs: sample (S), sample waste (SW), background electrolyte (BGE), sheath liquid (SL)

To evaluate ESI stability, the sheath liquid consisted of an organic solvent and milli-Q water (1:1, v/v) with 0.1% FA and 5 pmol/µL Glu-Fibrinopeptide B (GFP), loaded into the sheath liquid (SL) reservoir, and voltage was applied using a multichannel high-voltage power supply (µTK Microfluidic Tool Kit, Alberta Microelectronic Corporation) via a platinum electrode. ESI was initiated at voltages ranging from 2 to 3 kV, with a tip-to-inlet distance of approximately 1–2.5 mm. For sample injections and separation, potential differences were applied across the microchannels using the same system. To load the sample across the channel’s junction, the HVPS delivered +1 kV, ground connection, +0.7 kV, and +2.3 kV to the sample (S), sample waste (SW), BGE, and SL reservoirs, respectively (Fig. 1). This results in a pinch loading with approximately 100 pL sample volume. After ensuring that the loading channel was filled with the sample, the applied voltage was changed to +4.5, +4.5, +5, and +2.3 kV at the S, SW, BGE, and SL reservoirs, respectively, to initiate the separation. A 50:50 (v/v) mixture of MeOH and milli-Q water containing 0.1% FA was used as sheath liquid. The solution was spiked with GFP (5 pmol/µL), which served as the lock mass.

### Hemoglobin digestion

Human HB (13 mg) was dissolved in 0.23% FA to a final volume of 10 mL (21 μM). Immobilized pepsin beads (Thermo Scientific) were washed four times with 700 μL of 0.23% FA using a cellulose acetate spin filter (Corning® Costar® Spin-X® centrifuge tube filters). The HB solution was then incubated with the washed beads at ambient temperature with rocking for 18 h to allow proteolytic digestion. After digestion, the peptide solution was separated from the beads by centrifugation through a 0.45-μm cellulose acetate spin filter at 2000 × g for 2 min.

### Data acquisition and processing

Data were acquired using Xcalibur and Masslynx. Peptide identification by MS/MS was performed using a combination of both data-independent acquisition (DIA) and data-dependent acquisition (DDA). For DIA, the MS was run with a scan time of 0.3 s and collision cell switches at low and high energies of 6 and 25 V, respectively. DDA was performed using the top five intense precursor ions (≤ + 4 charge states) for fragmentation and MS/MS spectra were recorded with a scan time of 0.3 s and a TIC threshold of 500,000. All acquired mass spectra were corrected against GFP (lock mass) and identified using the ProteinLynx Global Server 3.0 (PLGS 3.0) database. The list of peptides identified by DDA was further validated by manual inspection of MS/MS spectra in PLGS 3.0. The list of peptides identified by DIA was filtered in DynamX 3.0 by a minimum of 0.3 products per amino acid and a maximum of 10 ppm precursor mass error. Sequence coverage maps were generated in DynamX 3.0 using the pooled list of peptides identified from either DDA or DIA by searching for each identified peptide in the respective MCE-MS data (using a 10-ppm precursor mass error tolerance and 20% RSD migration time (MT) tolerance and manually verifying the relative migration order of peptides).

## Results and discussion

### Microchip design and MS interfacing

The microchip was fabricated using a design similar to a prior report [54]. It had a cross design, featuring a 53-mm-long separation channel intersected by a 12-mm-long sample loading channel and an auxiliary side channel for introducing sheath liquid just before the integrated emitter, which provides both electrical circuit contact and ESI voltage. All microchannels had a height of 50 µm. The separation and sample channels were 50 µm wide, while the side channel was 200 µm wide. The separation channel ended in a sharp outlet defining the emitter tip that supported stable ESI (Fig. 1). There were four reservoirs for S, SW, BGE, and SL. The LTQ Orbitrap Velos was interfaced using a commercial Nanospray Flex ion source, in which glass cylinders were attached to the chip inlets and the chip was mounted directly onto the XYZ stage of the source (Fig. S1a). For the transition to the Waters Synapt G2-Si MS, a lab-built interface was used (Fig. S1b and c) to overcome geometry constraints associated with the ion source and improve flexibility in chip accessibility, positioning, alignment, and microscope imaging. The source was constructed around the Waters NanolockSpray XYZ stage and used a digital microscope (5-megapixel Dino-Lite) for monitoring ESI and chip alignment at the MS inlet. Chips were mounted between a milled PEEK holder and a PMMA support plate (Fig. S2). The PEEK holder contained four threaded inlet connections sealed to the chip inlets using O-rings. These threaded ports enabled direct connection to syringe pumps during coating procedures and channel washing between experiments. In addition, two alignment pins were incorporated into the holder design to ensure repeatable chip positioning.

### Evaluation of ESI stability

Experiments on uncoated TE chips were used to assess baseline ESI behavior and to identify limitations related to ESI stability and non-specific interactions. The observations provided the basis for evaluating the impact of heat treatment and subsequent surface coating strategies.

In preliminary tests, MeOH and ACN were evaluated as organic solvents (mixed with milli-Q water and FA) for on-chip ESI performance. ACN did not provide a stable ESI on the TE chip (Fig. S3a), whereas MeOH enabled repeatable ESI formation under identical conditions (Fig. S3b). Although the underlying mechanism was not investigated further, the observed difference is likely related to solvent dependent effects on Taylor cone formation and ESI stability. Across manufactured microchip replicates, ESI duration varied from a few minutes to over 30 min, likely due to differences in emitter geometry and surface quality. Degassing the solution using helium showed a slight improvement in stability; however, the effect was limited. This showed the importance of ESI stability and led to the use of thermal treatment in later fabrication steps.

### Heat treatment

To improve ESI stability and variability between chips, a post-fabrication oven heat treatment was applied to the TE microchips. The effect of heat treatment was evaluated by comparing ESI performance of untreated chips with those treated at 200 °C for 16 and 24 h, using the same BGE composition and emitter configuration across all tests. Chips treated for 24 h consistently maintained better stable ESI for at least 30 min, with minimal interruptions and no need for emitter repositioning. In contrast, the 16 h treatment and untreated chips yielded inconsistent results, frequently exhibiting short ESI durations and signal disruptions throughout the run (data not shown).

The improved performance observed after 24 h of heat treatment can be attributed to heat-induced changes in surface properties. Contact angle measurements (sessile water drop technique, Kruss drop shape analyzer DSA 100, Kruss Optronic, Hamburg, Germany) revealed a clear shift in surface wettability: untreated chips exhibited contact angles around 65°, while thermally treated chips reached approximately 85°, indicating increased hydrophobicity (Fig. S4). This effect is consistent with previously reported results on TE materials [42], which have shown that thermal treatment increases polymer density, raises the glass transition temperature, reduces solvent interaction, and improves surface uniformity. Although no obvious changes were observed in the emitter geometry, the surface properties following the heat treatment significantly improved ESI stability and emitter robustness. Based on these findings, heat treatment at 200 °C for 24 h was adopted as a standard preparation step for MCE chip fabrication.

### BGE optimization

The BGE composition was optimized in two steps: adjusting the FA concentration, followed by evaluating the MeOH percentage. All experiments in this section were performed using uncoated chips to assess the baseline separation performance under each condition. To achieve this, a mixture of small molecules and peptides was prepared: arginine, carnitine, ATII, BK, rhodamine B, LE, ME, and HEPES. Each compound was present at a concentration of 5 µM, except for rhodamine B, which was 500 nM. This mixture covered a molecular weight range from 162 to 1060 Da. Optimization was based on analyte detectability, MT repeatability, and overall peak shape.

**Fig. 2:**
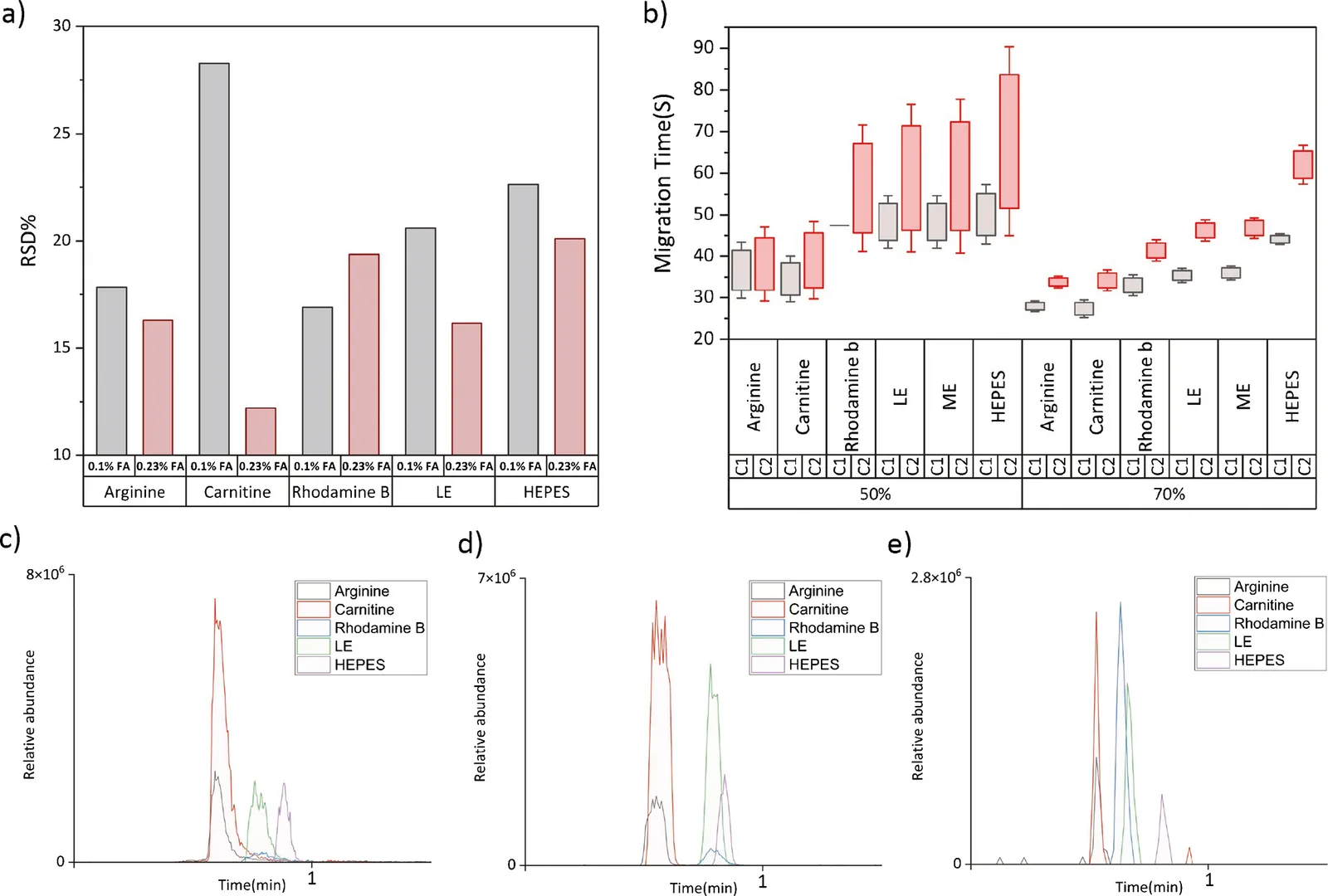
Investigating the impact of BGE during MCE-MS. **a** Comparison of MT repeatability (RSD%) across analytes using 0.1% (gray) and 0.23% (red) formic acid (FA) in the BGE (50% MeOH). *n* = 12, 3 chips. **b** Chip-to-chip comparison of MT at 50% and 70% MeOH concentration in the BGE (FA = 0.23%). *n* = 4, 2 chips (C1&C2). **c**, **d** EIEs related to the FA optimization (0.1% and 0.23%, respectively). **e** EIEs using 70% MeOH, 0.23% FA as BGE. EIEs represent the separation time (loading excluded)

Using 50% MeOH as the fixed organic content, the FA concentration in the BGE was evaluated at 0.1% and 0.23%. At 0.1% FA, most small molecules were detected; however, repeatability was poor with the MT RSD ranging from 17 to 22% (Fig. 2a). Larger peptides, such as BK and ATII, were detected in only a few replicates and showed poor consistency, indicating a high non-specific adsorption to the channel walls. LE, as a small peptide, was observed more reliably, with a MT RSD of 21%, though the peaks remained broad and asymmetric. An increase in FA concentration to 0.23% resulted in a moderate improvement in separation quality. MT repeatability improved for most analytes, with carnitine and LE showing RSDs of 12% and 16%, respectively. Detection of ATII and BK remained inconsistent, but overall peak shapes became slightly more defined (Fig. 2c and d). LE continued to serve as a representative performance indicator, with its MT RSD improving from 21% at 0.1% FA to 16% at 0.23% FA. Increasing the FA concentration from 0.1 to 0.23% primarily affected the repeatability of MT, with a limited influence on peak width. Full width half maximum (FWHM) values remained similar across both conditions. A higher concentration of FA could reduce local pH fluctuations that can otherwise destabilize EOF and increase variability in MTs [56]. In addition to improved pH stability, the increased ionic strength at 0.23% FA likely contributed to EOF stabilization by compressing the electrical double layer at the channel surface [56]. Based on these findings, 0.23% FA was selected as the suitable concentration for subsequent experiments.

Following the selection of 0.23% FA, the MeOH content in BGE was evaluated. While 50% MeOH was used in earlier optimization steps, further testing was conducted at 30% and 70%. At 30% MeOH, the high surface tension of the BGE prevented the formation of a stable Taylor cone, resulting in unstable ESI. Due to this, no reliable data could be collected using 30% MeOH. On the other hand, the best performance was obtained at 70% MeOH (Fig. 2b). On average, MTs decreased approximately 10% (up to 25%) compared to using 50% MeOH in the BGE. Nevertheless, the FWHM and shape were improved at the higher percentage of organic solvent (Fig. 2e). FWHM values were consistently lower across all analytes, with several compounds showing a reduction exceeding 50% compared to lower MeOH content BGE. Peaks became more symmetrical, indicating a decrease in wall interaction. Arginine and rhodamine B also showed improved peak shape, exhibiting a sharp decrease in MT RSD%.

This improvement is likely not only due to increased analyte mobility but also to enhanced ESI performance. Higher MeOH content could influence surface tension, enabling more stable Taylor cone formation and finer initial droplet size, which could improve desolvation and ionization efficiency [57]. Based on the improved FWHM and peak shapes, especially for peptides, 70% MeOH as a BGE modifier was used for all subsequent experiments.

### Coating of the MCE channel

The effect of surface modification of the MCE channel on separation performance was investigated by comparing three coatings: mPEG, LPA, and PEGMA. Neutral hydrophilic polymer coatings shield the charged surface groups responsible for generating EOF in CE. Additionally, shielding of the surface functional groups reduces analyte adsorption to the channel surface [58, 59]. One advantage of the TE material is the presence of residual thiol and allyl(ene) functionalities on the surface that enable post-fabrication surface modification. A previous study has utilized these reactive groups to covalently anchor neutral polyacrylate-based coatings onto the excess thiol in the TE surface [52]. In the present work, mPEG was linked to ene groups on the surface after first adding primary amine functional groups (cysteamine), while LPA and PEGMA coatings were immobilized via click chemistry to the TE surface thiol groups.

All experiments were performed on microchips subjected to 24-h heat treatment at 200 °C and operated under optimized BGE conditions (70% MeOH, 0.23% FA). Performance evaluation focused on MT repeatability, FWHM, and analyte detectability across mixtures of peptides.

### mPEG coating

The mPEG coating involved a two-step procedure. First, cysteamine was immobilized on the TE surface to introduce primary amine groups. These were then reacted with mPEG-NHS to form covalent amide bonds; this modification aimed to reduce peptide adsorption and eliminate EOF. The coating was successfully implemented on only a single microchip due to significant challenges encountered during the procedure. The incubation step frequently led to premature solidification, resulting in partial or complete clogging of the microchannels. As a result, no chip-to-chip evaluation was performed. Nevertheless, the coating provided complete analyte detection with acceptable separation performance (*n* = 4) (Fig. S7a). All target compounds (except HEPES), including large peptides such as BK and ATII, were detected with consistent MT. MT RSDs ranged from 9% for BK to 33% for rhodamine B. FWHM values were between 2.8 and 5.7 s for small molecules and moderate-sized peptides. The larger peptides, BK and ATII, showed broader peaks (8.6 and 8.5 s, respectively).

While the coating demonstrated reliable performance in terms of analyte detectability and peak shape on a functional chip, the inability to replicate the coating process across multiple devices presents a significant limitation. The data support that the chemical strategy can be effective when implemented successfully, but the overall utility of the mPEG coating is limited by low fabrication repeatability.

### LPA coating

LPA was selected for coating due to its known capacity to suppress EOF and minimize non-specific adsorption of proteins and peptides in electromigration separations [60]. LPA was grafted onto TE surfaces via UV-initiated polymerization, using TPO-L as the photoinitiator. The resulting layer forms a neutral hydrophilic coating on the channel surface. This coating was applied in multiple cycles to improve uniformity and hydrophilicity, and also minimize the effect of homopolymerization that can limit effective surface grafting. The LPA coating provided sharp peaks during the separation of both small molecules and low-molecular-weight peptides (Fig. S7b). FWHM values for most compounds were typically between 1.4 and 2.5 s, while repeatability was moderate, with RSD of 26–31%.

**Fig. 3:**
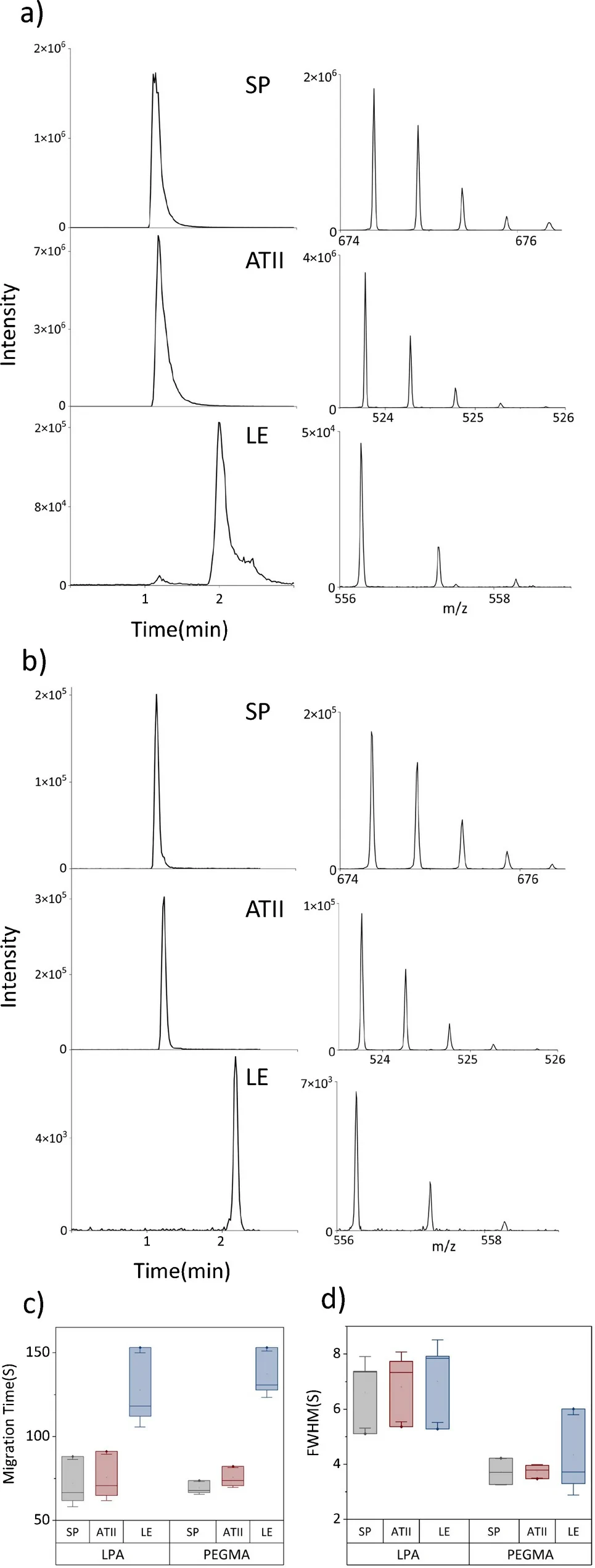
Effect of coating on MCE-MS separation of model peptides (SP, ATII, and LE). **a** EIEs using a LPA-coated microchip and corresponding mass spectra. **b** EIEs using a PEGMA-coated microchip and corresponding mass spectra. **c**, **d** Comparison of the effect of coating on MT and FWHM, *n* = 3. EIEs represent the separation time (loading excluded)

Repeatability of MT was also satisfactory, with RSD below 12% for all reliably detected analytes. Our results align well with results from other CE studies in non-chip formats that show that LPA coatings significantly suppress EOF and improve peak shape while minimizing adsorption, even in the presence of organic solvents [61]. Chip-to-chip comparisons showed comparable trends, indicating a consistent coating process across replicates and good inter-chip repeatability. The main drawback was the inability to detect larger peptides under these conditions. Similar results were obtained upon interfacing the MCE-MS chip to a more sensitive MS instrument (Synapt G2-Si). To further evaluate the detection of larger peptides using the LPA coating, a sample mixture of SP, ATII, and LE was analyzed, in which the concentration of the larger peptides was increased (20 µM for SP and ATII and 5 µM for LE). All three peptides were detected at these concentrations (Fig. 3a), with FWHMs of approximately 7 s. The MT RSD% values were 10%, 12%, and 19% for ATII, LE, and SP, respectively (*n* = 3), with theoretical plate numbers up to 68 × 10^3/m for LE.

### PEGMA coating

PEGMA was covalently attached to thiol-functionalized surfaces through UV-initiated TE click chemistry. Under UV light, free radicals formed from the initiator abstracted hydrogen from thiol groups, generating thiyl radicals that then bonded across the vinyl groups in PEGMA. This reaction formed stable thiol-carbon bonds, creating a PEG-based coating layer. PEGMA was chosen for its capacity to reduce EOF while maintaining surface hydrophilicity [62]. PEGMA-coated microchips were prepared using repeated UV exposure cycles to improve surface coverage and uniformity. Multiple cycles were necessary because PEGMA can undergo homopolymerization, which can limit effective surface grafting during a single exposure. The coating enabled detection of all target analytes across three independently prepared chips (Fig. S7c). MT RSDs were below 11%. Additionally, chip-to-chip consistency for MT was preserved. Larger peptides, such as BK and ATII, were also consistently detected, although the ATII signal exhibited extended tailing and a highly broadened profile.

An identical experiment, using a mixture of selected peptides (SP, ATII, LE) at higher concentrations, was conducted by interfacing the MCE-MS device to a more sensitive MS using a custom-made PEEK holder (Fig. 3b). The peak shapes were predominantly symmetrical, with the FWHM in the range of 3.7 to 4.3 s. MT% RSDs ranged from 5 to 10% for all three peptides across three replicate injections. The number of theoretical plates was 36 × 10^3/m, 43 × 10^3/m, and 104 × 10^3/m for SP, ATII, and LE, respectively. The somewhat improved performance observed with PEGMA could be linked to its nonlinear nature as opposed to the linear polyacrylamide. PEGMA forms a dense, brush-like hydrophilic surface structure, which may more effectively suppress EOF and minimize unspecific peptide-channel wall interactions [63, 64].

Overall, the evaluated coatings showed distinct advantages for peptide separations on TE microchips. The mPEG coating achieved acceptable separation performance but suffered from poor fabrication repeatability, attributable to channel clogging during the coating process. LPA coating produced sharp peaks and repeatable MTs, with additional experiments with higher peptide concentrations confirming compatibility with larger peptides. PEGMA coating demonstrated the most balanced overall performance, offering sharp peaks, broad analyte detectability, and repeatable MTs across microchips. Consequently, both LPA and PEGMA coatings were selected for subsequent evaluation in peptide mapping MCE-MS/MS experiments.

### Peptide mapping applications of the MCE-MS chip

**Fig. 4:**
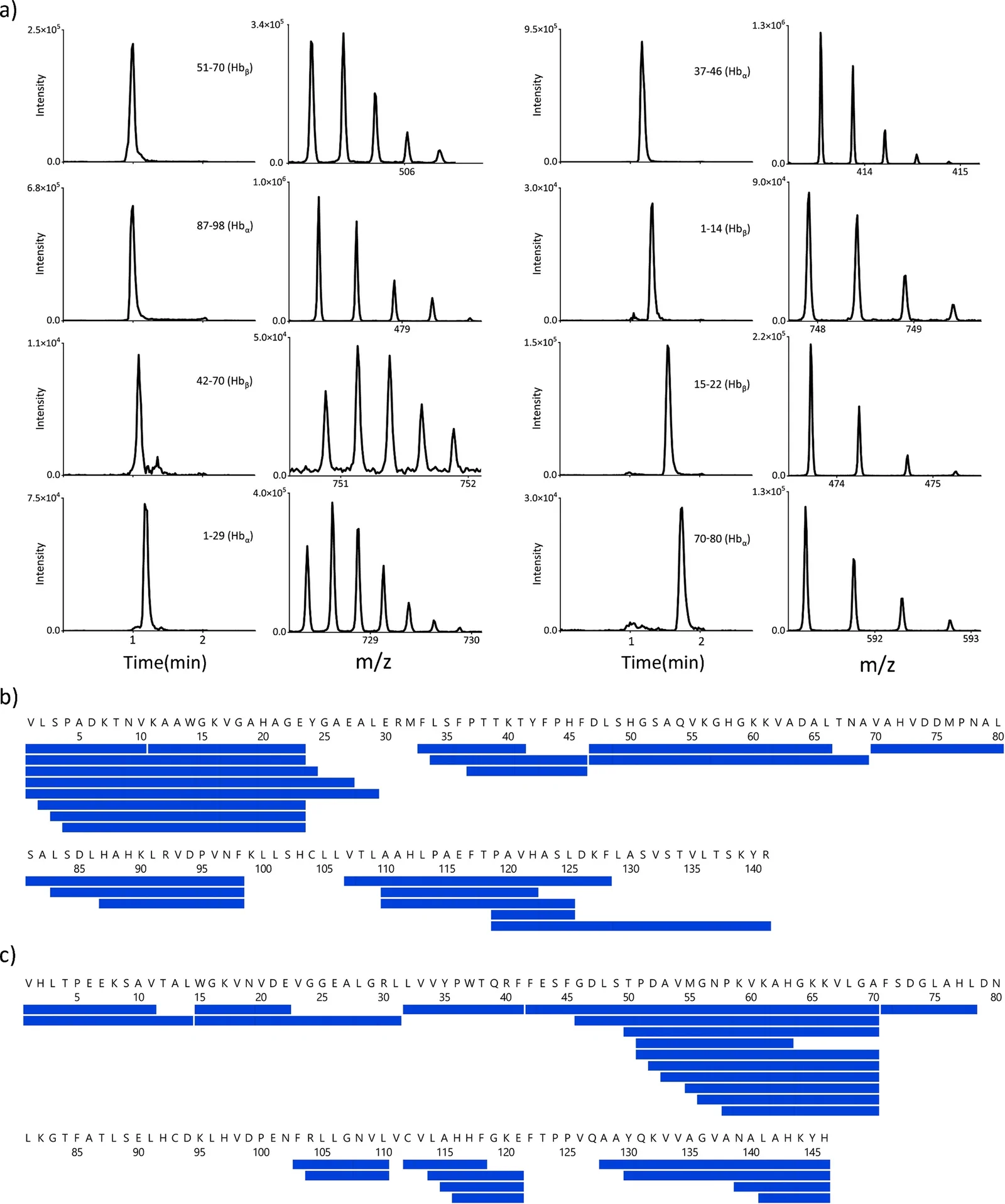
Peptide mapping MCE-MS analysis using the PEGMA-coated TE-based microchip. **a** EIEs and related mass spectra from MCE-MS analysis of the peptic digest of HB. **b** Coverage map of the HB α-chain. **c** Coverage map of the HB β-chain. EIEs represent the separation time (loading excluded)

LPA- and PEGMA-coated microchips were used for peptide mapping MCE-MS/MS analysis of the complex mixture of peptides resulting from pepsin digestion of human HB. Both coatings enabled the MS/MS identification of peptides from α- and β-chains within a short separation window. Representative extracted electropherograms (EIEs) and MS spectra are shown in Fig. 4a using the PEGMA-coated chip and Fig. S8a for the LPA-coated microchip.

Using PEGMA microchips, peptide migration occurred within a 60-s separation window, with an average FWHM of HB peptides of 4.1 s. In total, 23 peptides were assigned to the α-chain and 26 to the β-chain, resulting in a sequence coverage of 92.2% and 78.8%, respectively (Fig. 4b and c). The corresponding redundancies were 3.09 for the α-chain and 3.04 for the β-chain. The absence of peptide coverage in regions containing cysteine residues may be related to oxidation sites in HB (Hbβ-Cys93), which has previously been reported [65]. Chip-to-chip comparison using full-scan MS data from two independently prepared devices showed good repeatability with MT RSDs less than 6% across the top 10 most intense peptides.

LPA-coated microchips also enabled comparable peptide identifications, although slightly lower sequence coverage was obtained compared to that on the PEGMA-coated microchip at 85.8% and 77.4%, respectively, for the α-chain and β-chain (Fig. S8b and c). A total of 15 and 24 peptides were identified for α-chain and β-chain, respectively, within a similar separation window, with an average FWHM of 6.6 s.

Overall, coating the MCE channel of the TE microchip with either PEGMA or LPA enabled efficient peptide mapping MCE-MS/MS analysis. However, PEGMA-coated chips provided the most favorable balance between sequence coverage and peak shape under the tested conditions.

### MCE-MS of intact proteins

The performance of the PEGMA-coated microchip for intact protein analysis was evaluated using a mixture of insulin, β-lactoglobulin, and tropomyosin, each at 10 µM. Protein samples were prepared in 30% MeOH with 0.1% FA. Separations were performed using a BGE of 70% MeOH and 0.23% FA. All experiments were conducted under consistent separation conditions.

**Fig. 5:**
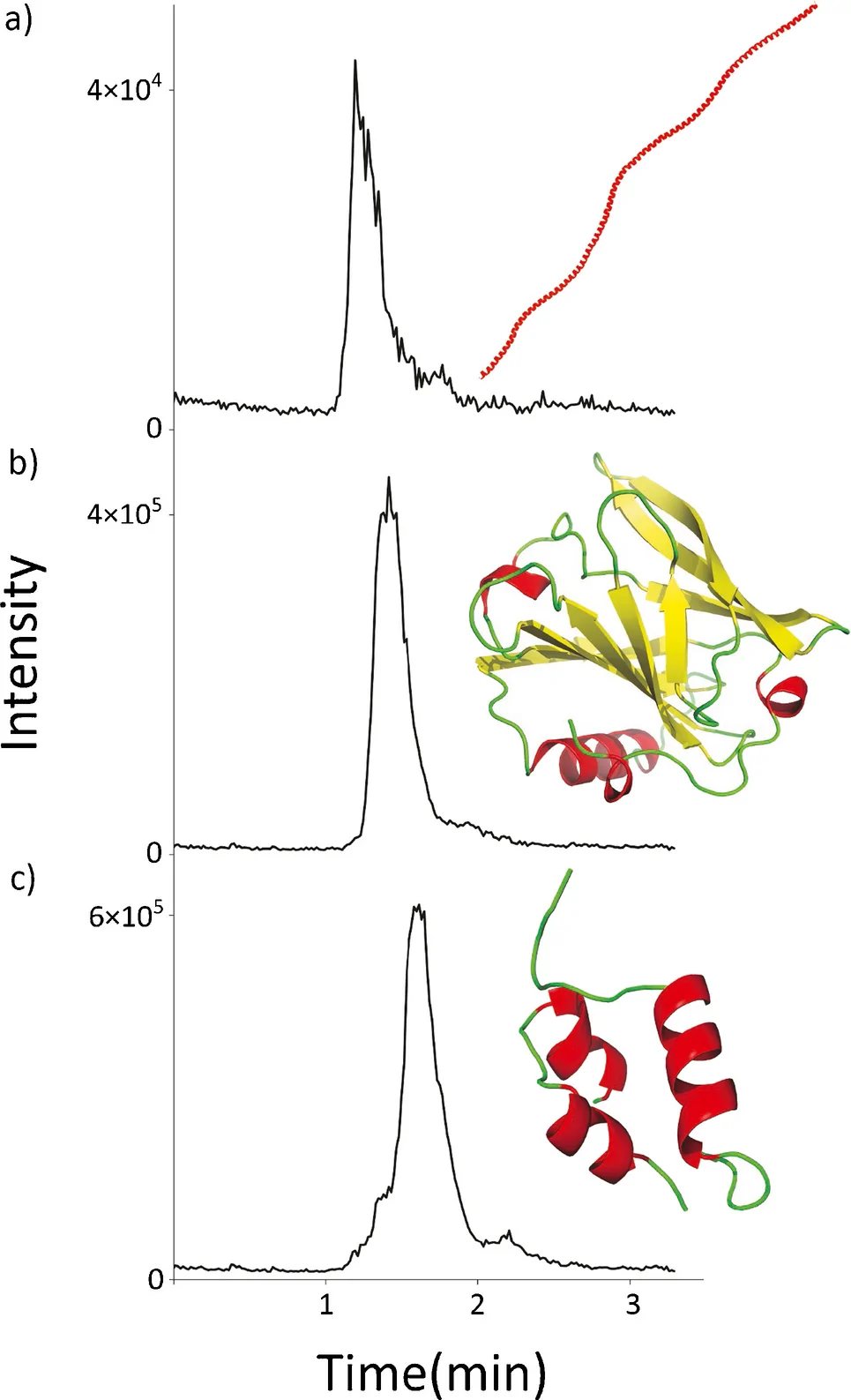
Intact protein MCE-MS analysis using the PEGMA-coated TE-based microchip. **a** EIE of tropomyosin, mass, 32.7 kDa. **b** EIE of β-lactoglobulin, mass, 18.2 kDa. **c** EIE of insulin, mass, 5.7 kDa. EIEs represent the separation time (loading excluded)

Distinct protein signals were detected within a short migration window, with FWHM under 20 s. Tropomyosin, β-lactoglobulin, and insulin migrated with average MTs of 77 s, 86 s, and 99 s, respectively (Fig. 5). The observed migration order reflects differences in electrophoretic mobility, as there is not expected to be a significant EOF (if any) after coating. The electrophoretic mobility of a protein is related to the protein charge-to-size ratio. At approximately pH 2.5, protonation of basic residues such as lysine and arginine substantially increases the net positive charge of the proteins, causing all three proteins to carry a substantial net positive charge (the pI of all three proteins is in the same range, around 4.5–5.5). The observed difference in migration is primarily caused by differences in size, i.e., mass and conformation at low pH. If all three proteins were fully unfolded at the low pH of the BGE, one would thus expect the migration order to be insulin (5.7 kDa), β-lactoglobulin (18.2 kDa), and tropomyosin (32.7 kDa). However, tropomyosin is observed to migrate fastest, which could indicate an ability of the protein to form a very compact helical form at low pH. Oppositely, insulin migrates the slowest despite having the smallest mass of the three proteins, possibly due to the presence of S-S bonds of insulin keeping it in an extended structure even at the low pH. These MTs align well with another study using conventional CE [66].

MT repeatability was assessed using two replicate runs. RSDs less than 2% were obtained for all proteins. These results indicate repeatable MTs and support the suitability of the PEGMA-coated microchip for intact protein analysis.

## Conclusion

Here, we have developed a TE-based MCE-MS microchip with an integrated ESI emitter for peptide and protein analysis. We assessed the impact of device fabrication, MS interfacing, separation conditions, and surface modification strategies on the performance of the microchip. Under optimal conditions, stable ESI and repeatable electrophoretic separations were achieved, enabling the analysis of both peptides and intact proteins. Among the evaluated surface coatings, PEGMA offers the most balanced overall performance, combining broad analyte compatibility, repeatable MTs, and repeatable peptide and protein mass analysis. LPA coating also demonstrated favorable separation efficiency and repeatability, although with lower compatibility toward larger peptides. Using the optimized coated MCE-MS microchips, peptide mapping of pepsin-digested HB was performed with high sequence coverage within a short electrophoretic separation window of 60 s. In addition, the MCE-MS device was capable of separation and mass analysis of intact proteins under acidic CE-MS conditions, highlighting also the application of the chip for mass analysis of intact proteins in a mixture.

Overall, the results demonstrate the potential of TE microchips as a robust and versatile platform for MCE-MS analysis of peptides and proteins. The combination of integrated ESI, short analysis times, and adaptable surface chemistry provides a promising basis for future development of TE-based MCE-MS for a variety of bioanalytical applications, workflows, and applications.

## Supplementary Information

Below is the link to the electronic supplementary material.

Supplementary file1 (DOCX 8.07 MB)

## Data Availability

Data will be made available on request.
