# Supplementary material for: Development of a thiol-ene microfluidic chip for CE-MS of peptides and proteins
Source: Anal Bioanal Chem. 2026 Sep 10;418(22):7301–14. doi: 10.1007/s00216-026-06778-7 (PMC13736354; doi:10.1007/s00216-026-06778-7)
Supplement: Supplementary file 1 [file 216_2026_6778_MOESM1_ESM.docx]

**Development of a thiol-ene microfluidic chip for CE-MS of peptides and proteins**

Omid Rouhi^a^, Jordan T. Aerts^a^, Kamille C. Staack^a^, Delaram R. Mikalsen^a^, Stephan S. Keller^c^, Mohammad Ramezannezhad^c^, Nickolaj J. Petersen^b^, Jörg P. Kutter^b^, and Kasper D. Rand^a^*.

^a^ Protein Analysis Group, Department of Pharmacy, University of Copenhagen, 2100 Copenhagen O, Denmark
^b^ Pharmaceutical Analytical Chemistry, Department of Pharmacy, University of Copenhagen, 2100 Copenhagen O, Denmark

^c^ National Centre for Nano Fabrication and Characterization, DTU Nanolab, Technical University of Denmark, 2800 Kgs. Lyngby, DK, Denmark

* To whom correspondence may be addressed: Dr. Kasper Rand, email: [kasper.rand@sund.ku.dk](mailto:kasper.rand@sund.ku.dk)

**Fig. S1** **Microfluidic capillary electrophoresis mass spectrometry (MSE-MS) interface**. a) Commercial ThermoFisher Nanospray flex interface coupled to LTQ Orbitrap Velos. b) MCE-MS experiment setup using Waters Synapt G2-Si MS. c) Customized lab-built interface

**Fig. S2 PEEK holder and a PMMA support plate for microchip coupled to Waters Synapt G2-Si MS.** Threaded inlet port for direct connection to syringe pump(top) and sealing to the microchip inlets using O-rings(down)

**Fig. S3 Evaluation of electrospray ionization (ESI) stability.** a) Acetonitrile as the organic solvent. b) Methanol as the organic solvent. c) Glu-fibrinopeptide B (GFP)mass spectrum. Sheath liquid consisted of an organic solvent and milli-Q water (1:1, v/v) with 0.1% formic acid (FA) spiked with 5 pmol/µL (GFP), loaded into the side arm reservoir. TIE: total ion electropherogram, EIE: extracted ion electropherogram.

**Fig. S4 The effect of heat treatment on the thiol-ene(TE) surface contact angle.** a) Water droplet on the surface before heat treatment. b) Water droplet on the surface after heat treatment. c) Contact angle comparison.

**Fig. S 5 The effect of coating on TE surface contact angle.** a) PEGMA-coated heat-treated TE contact angle 47° (2.3% RSD) n=3. b) LPA coated heat-treated TE contact angle 32° (2.0% RSD). LPA was shown after only four of five cycles due to difficulty seeing the droplet after completing all five coating cycles. Contact angle after all five cycles <10%

**Fig. S6 Rhodamine b migration time comparison across surface coatings utilizing MCE-UV.** Microchip dimensions and geometry were as follows: all channels were 50 µm wide and 50 µm deep. Distances from the reservoirs, sample, waste, and background electrolyte to the junction were 7 mm, and the separation channel length was 4.2 cm. Migration times were compared across surface coatings (Uncoated, LPA, mPEG, and PEGMA) using two chips per type, with three injections per chip. All measurements were performed using fluorescence microscopy

**Fig. S7 The effect of coating on MCE-MS using a mixture of small molecules and peptides.** a) EIEs using a mPEG-coated chip. b) EIEs using an LPA-coated chip. c) EIEs using a PEGMA-coated chip. d) Comparison of the effect of coating on migration time, n=3. EIEs represent the separation time (loading excluded)

**Fig. S8 Peptide mapping MCE-MS analysis using the LPA-coated TE-based microchip**. a) EIEs and related mass spectra from MCE-MS analysis of the peptic digest of hemoglobin. b) Coverage map of the HB α-chain. c) Coverage map of the HB β-chain. EIEs represent the separation time (loading excluded)

Fig. S9 **Mass spectra (left panels) and corresponding MAXEnt1 deconvoluted spectra (right panels) for Intact protein MCE-MS analysis using the PEGMA-coated TE-based microchip**. a, b) Tropomyosin (UniProt p42639), mass: 32735.7 Da[1], mass error 13ppm. c, d) β-lactoglobulin (UniProt p02754), mass:18277.7 Da, mass error: 59ppm. e, f) Insulin (UniProt p01317), mass: 5733.5 Da, mass error: 69ppm. Mass spectra were subtracted to exclude the lock mass spectrum (GFP, m/z: 785.84)

1.	Peng, Y., et al., *Top-down targeted proteomics for deep sequencing of tropomyosin isoforms.* Journal of proteome research, 2013. **12**(1): p. 187-198.
